# Supplementary material for: The 2EF Antibody Targets a Unique N-Terminal Epitope of Trop-2 and Enhances the In Vivo Activity of the Cancer-Selective 2G10 Antibody
Source: Cancers (Basel). 2023 Jul 22;15(14):3721. doi: 10.3390/cancers15143721 (PMC10378344; doi:10.3390/cancers15143721)
Supplement: Supplementary file 1 [file cancers-15-03721-s001.zip › cancers-2521282-supplementary.pdf]

SUPPLEMENTARY ONLINE MATERIAL

Supplementary Methods

Cells. The human MCF-7 and MDA-MB231 breast cancer, DU145 prostate cancer, SKOv3 ovarian cancer, Colo205 and HT29 colon cancer were obtained from the American Type Culture Collection (ATCC). The murine L fibrosarcoma cell line [1] was a kind gift by Leonard A. Herzenberg (Stanford University, CA). The HCT-116, HCT-116 U5.5 and KM12SM cell lines were obtained from Roche Diagnostics GmbH, Pharma Research (Penzberg, Germany). The KM12SM cell line was derived from KM12 hepatic metastases as described [2]. The murine immortalized MTE 4-14 thymic cell line was a kind gift by Philippe Naquet, and was established and characterized as described [3]. The NS-0 murine myeloma was obtained from the European Collection of Authenticated Cell Cultures (ECACC, Salisbury, Wiltshire, UK). The YB2/0 fucosylation-less rat myeloma [4] was obtained from the American Type Culture Collection (ATCC, Manassas, VA). The human 293T kidney cell line was obtained from Angelo Veronese (University 'G. d'Annunzio', Chieti, Italy). Frozen master stocks were prepared for each cell line within the first two months from receipt. Experiments were performed on cells that had been resuscitated from master stocks and passaged for no more than 6 months. Cell cultures were periodically checked for absence of mycoplasma contamination by PCR analyses and DAPI staining. The MCF-7, MDA-MB231, DU145, SKOv3, Colo205 and HT29 cell lines were grown in RPMI 1640 medium. KM12SM cells line were grown in RPMI 1640 medium supplemented with non-essential amino acids and MEM vitamins (Euroclone, Milano, Italy). The 293T, HCT-116, HCT-116 U5.5, MTE4-14, NS-0, YB2/0 and L cell lines were maintained in DMEM medium supplemented with 10% FCS. All media were supplemented with 10% fetal calf serum (FCS), 100 IU/ml penicillin and 100 μg/ml streptomycin (Euroclone, Milano, Italy).

Antibodies. The AbT16 [5,6], 2G10 [7,8], 2EF, AR47A6.4.2 [9] anti-Trop-2 monoclonal antibodies (mAb) were purified and fluorochrome-conjugated as described [1].

Endotoxin measurement. Contaminating endotoxin in antibody solutions and reagents was measured with the *Limulus Amebocyte* lysate (LAL) test (International PBI, Milano, Italy). The reagents used for the *in-vivo* experiments scored negative at the highest sensitivity used, i.e. endotoxin < 0.03 U/ml (= 0,006 ng/ml).

Cell-growth assays. HT29 transfectants were seeded at 1.5-3.0 $\times 10^3$ cells/well (low density) or 10-15 $\times 10^3$ cells/well (high density) in 96-well plates (five replica wells per data point). Cell numbers were quantified by staining with crystal violet [3]. Cell numbers were normalized against standard

reference curves of two-fold serially diluted cell samples. Cell numbers were quantified in parallel by image analysis/nuclei count of DAPI-stained 96 well plates.

Figure legends

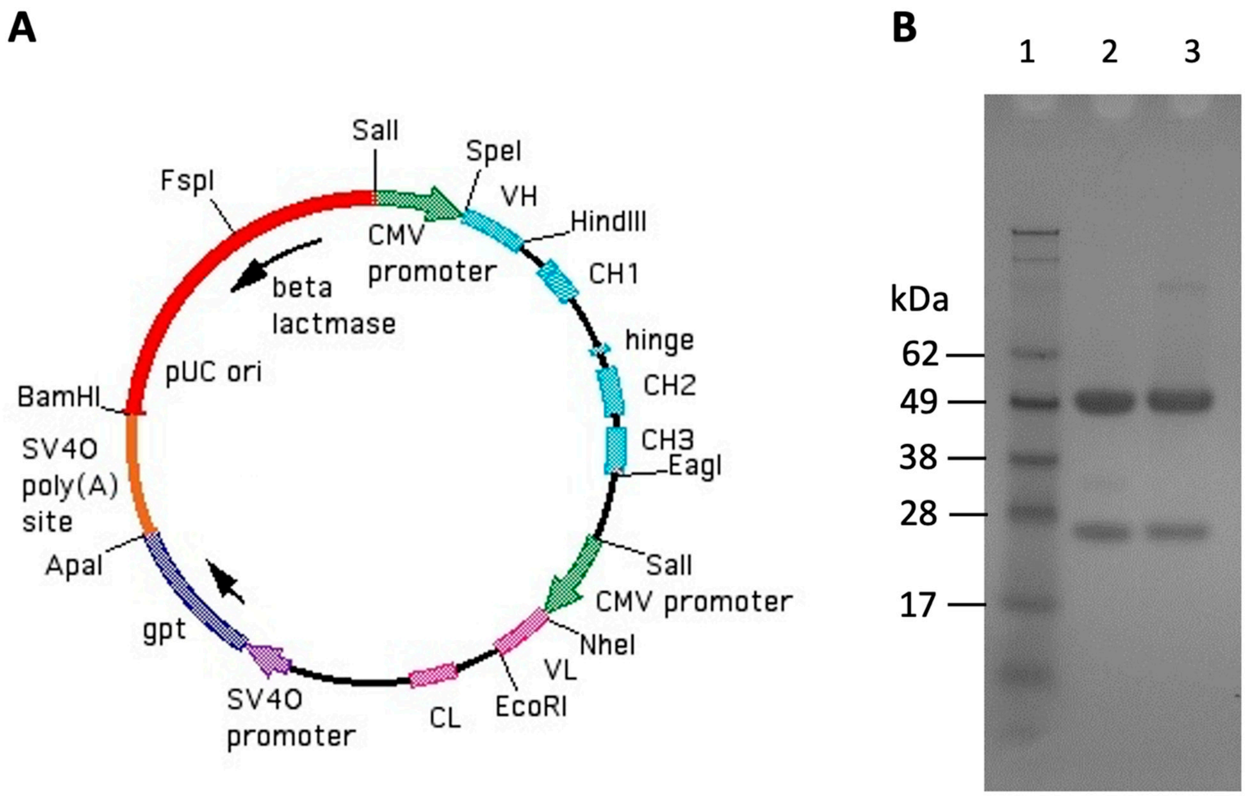

Supplementary Figure S1. (**A**) Expression vector for the chimeric and humanized 2EF. The heavy chain transcription unit starts with the human cytomegalovirus (CMV) major immediate early promoter and enhancer (CMV promoter). The CMV promoter is followed by the VH exon, a genomic sequence containing the human gamma-1 heavy chain constant region including the CH1, hinge, CH2 and CH3 exons with the intervening introns, and the polyadenylation site following the CH3 exon. The light chain transcription unit begins with the CMV promoter, followed by the VL exon and a genomic sequence containing the human kappa chain constant region exon (CL) with part of the intron preceding it and the polyadenylation site following the CL exon. The light chain gene is then followed by the SV40 early promoter, the *E. coli* xanthine guanine phosphoribosyl transferase gene (gpt), and the SV40 polyadenylation site. In pHu2EF-7-puro the gpt gene was replaced by the puromycin N-acetyltransferase gene. (**B**) SDS PAGE analysis of Hu2EF-7 antibodies. Antibodies (5 μg each) were run on a 4-20% SDS PAGE gel, under reducing conditions and stained with SimplyBlue Safestain. Lane 1, SeeBluePlus2 prestained molecular weight standards; lane 2, Hu2EF-7 purified from CHO-K1-Hu2EF-7 2A2.2 cells; lane 3, Hu2EF-7 purified from YB2/0-Hu2EF-7 2D3 cells.

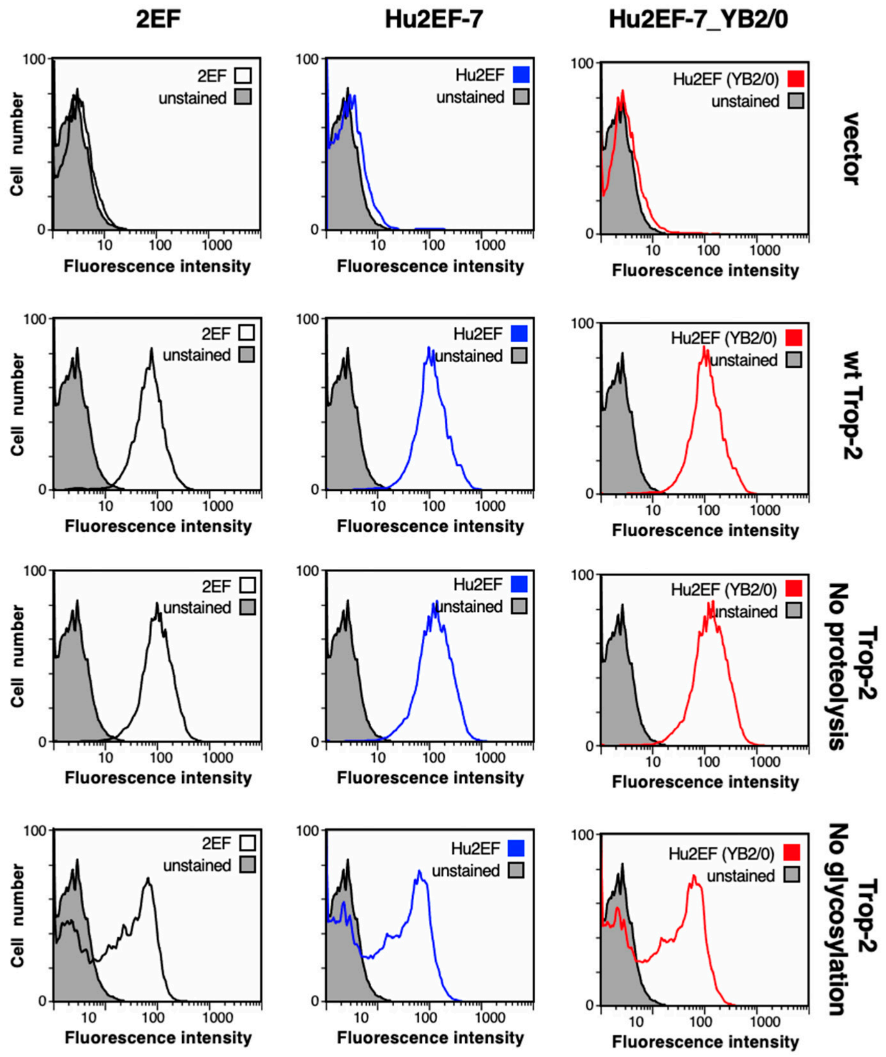

Supplementary Figure S2. Flow cytometry analysis of engineered Hu2EF binding to target KM12SM/Trop-2 transfected cells [2,3]. Vector: control KM12SM cells, transfected with vector alone. wtTrop-2: KM12SM cells, transfected with wtTrop-2. Trop-2 no-proteolysis: KM12SM cells, transfected with Trop-2 mutagenized at the ADAM10 cleavage site. Trop-2 no-glycosylation: KM12SM cells, transfected with Trop-2 mutagenized at its four glycosylation sites. 2EF: Mouse 2EF-22.21-Alexa488; Hu2EF: Hu2EF-7-Alexa488; Hu2EF (YB2/0): Hu2EF-7-Alexa488 produced in fucosylation-defective YB2/0 cells [4].

Supplementary references

1. Alberti, S.; Nutini, M.; Herzenberg, L.A. DNA methylation prevents the amplification of TROP1, a tumor associated cell surface antigen gene. *Proc. Natl. Acad. Sci. USA* **1994**, *91*, 5833-5837.
2. Guerra, E.; Trerotola, M.; Relli, V.; Lattanzio, R.; Tripaldi, R.; Vacca, G.; Ceci, M.; Boujnah, K.; Garbo, V.; Moschella, A.; et al. Trop-2 induces ADAM10-mediated cleavage of E-cadherin and drives EMT-less metastasis in colon cancer. *Neoplasia* **2021**, *23*, 898-911, doi:https://doi.org/10.1016/j.neo.2021.07.002.
3. Trerotola, M.; Guerra, E.; Ali, Z.; Aloisi, A.L.; Ceci, M.; Simeone, P.; Acciarito, A.; Zanna, P.; Vacca, G.; D'Amore, A.; et al. Trop-2 cleavage by ADAM10 is an activator switch for cancer growth and metastasis. *Neoplasia* **2021**, *23*, 415-428, doi:10.1016/j.neo.2021.03.006.
4. Teylaert, B.; Meurice, E.; Bobowski, M.; Harduin-Lepers, A.; Gaucher, C.; Fontayne, A.; Jorieux, S.; Delannoy, P. Molecular cloning, characterization, genomic organization and promoter analysis of the α1,6-fucosyltransferase gene (fut8) expressed in the rat hybridoma cell line YB2/0. *BMC Biotechnology* **2011**, *11*, 1, doi:10.1186/1472-6750-11-1.
5. Fradet, Y.; Cordon-Cardo, C.; Thomson, T.; Daly, M.E.; Whitmore Jr, W.F.; Lloyd, K.O.; Melamed, M.R.; Old, L.G. Cell-surface antigens of human bladder cancer defined by mouse monoclonal antibodies. *Proc. Natl. Acad. Sci. USA* **1984**, *81*, 224-228.
6. Alberti, S.; Miotti, S.; Stella, M.; Klein, C.E.; Fornaro, M.; Ménard, S.; Colnaghi, M.I. Biochemical characterization of Trop-2, a cell surface molecule expressed by human carcinomas: formal proof that the monoclonal antibodies T16 and MOv-16 recognize Trop-2. *Hybridoma* **1992**, *11*, 539-535.
7. Alberti, S.; Trerotola, M.; Guerra, E. The Hu2G10 mAb targets the cleaved-activated form of Trop-2 and exploits vulnerability of multiple human cancers. *Journal of Clinical Oncology* **2022**, *40*, e14548-e14548, doi:10.1200/JCO.2022.40.16_suppl.e14548.
8. Alberti, S.; Trerotola, M.; Guerra, E. Abstract 340: The Hu2G10 tumor-selective anti-Trop-2 monoclonal antibody targets the cleaved-activated Trop-2 and shows therapeutic efficacy against multiple human cancers. *Cancer Research* **2022**, *82*, 340-340, doi:10.1158/1538-7445.AM2022-340.
9. Truong, A.H.L.; Feng, N.; Sayegh, D.; Mak, B.C.; O'Reilly, K.; Fung, S.W.; Ceric, N.; Hahn, S.H.; Pereira, D.; Findlay, H. AR47A6.4.2, a functional naked monoclonal antibody targeting Trop-2, demonstrates in vivo efficacy in human pancreatic, colon, breast and prostate cancer models. *Molecular Cancer Therapeutics* **2007**, *6*, 3334s-3334s.
